# A New Inovirus from the Human Blood Encodes Proteins with Nuclear Subcellular Localization

**DOI:** 10.3390/v16030475

**Published:** 2024-03-20

**Authors:** Nikolay Popgeorgiev, Mart Krupovic, Julien Hiblot, Laura Fancello, Sonia Monteil-Bouchard, Christelle Desnues

**Affiliations:** 1 Université de Lyon, Centre de Recherche en Cancérologie de Lyon, U1052 INSERM, UMR CNRS 5286, Université Lyon I, Centre Léon Bérard, 28 rue Laennec, 69008 Lyon, France; 2 Institut Universitaire de France (IUF), 75013 Paris, France; 3 Archaeal Virology Unit, Institut Pasteur, Université Paris Cité, 75015 Paris, France; 4 Department of Chemical Biology, Max Planck Institute for Medical Research, Jahnstrasse 29, 69120 Heidelberg, Germany; julien.hiblot@mr.mpg.de; 5 Interdisciplinary Research Institute of Grenoble, IRIG-Biosanté, University Grenoble Alpes, CEA, INSERM, UMR 1292, 38000 Grenoble, France; laura.fancello@gmail.com; 6 Microbiologie Environnementale Biotechnologie, Institut Méditerranéen d’Océanologie, 163 Avenue de Luminy, 13009 Marseille, France; sonia.monteil@univ-amu.fr (S.M.-B.); christelle.desnues@univ-amu.fr (C.D.)

**Keywords:** inoviruses, metagenomics, nuclear localization

## Abstract

Viruses infecting bacteria (bacteriophages) represent the most abundant viral particles in the human body. They participate in the control of the human-associated bacterial communities and play an important role in the dissemination of virulence genes. Here, we present the identification of a new filamentous single-stranded DNA phage of the family *Inoviridae*, named Ralstonia Inoviridae Phage 1 (RIP1), in the human blood. Metagenomics and PCR analyses detected the RIP1 genome in blood serum, in the absence of concomitant bacterial infection or contamination, suggesting inovirus persistence in the human blood. Finally, we have experimentally demonstrated that the RIP1-encoded rolling circle replication initiation protein and serine integrase have functional nuclear localization signals and upon expression in eukaryotic cells both proteins were translocated into the nucleus. This observation adds to the growing body of data suggesting that phages could have an overlooked impact on the evolution of eukaryotic cells.

## 1. Introduction

The human body is home to diverse viral flora [1,2]. Viruses that infect bacteria, also known as bacteriophages, are the most abundant and diverse viral entities in the human body [3]. Bacteriophages are detected in virtually all anatomical sites, being most abundant in the digestive system [4], the respiratory tract [5], and on the skin [6]. Phage communities play a critical role in the control of the bacterial populations in humans. More recently, bacteriophages have also emerged as potential virulence gene carriers, which can participate in bacterial pathogenicity through lateral gene transfer. Seminal observations in viral metagenomes of the oral cavity of healthy individuals, as well as cystic fibrosis patients, showed that phages represent an important reservoir for bacterial virulence and resistance genes, thus contributing to bacterial pathogenicity [5,7,8].

Filamentous bacteriophages of the order *Tubulavirales* have emerged as a highly diverse group of viruses, globally distributed across biomes and infecting a broad range of bacterial hosts [9]. Based on comparative genomics analysis and the host range, members of the *Tubulavirales* are divided into three families, *Inoviridae*, *Plectroviridae*, and *Paulinoviridae*, but many family-level groups await formal classification [9,10]. Filamentous phages infecting Gram-negative bacteria have been studied most extensively and belong to the family *Inoviridae* [11,12]. Inoviruses have slender flexible virions, usually about 6–10 nm in diameter and 600–2500 nm long [12,13]. Inovirus genomes consist of circular single-stranded DNA (ssDNA) molecules of about 5.5–10.6 kb, and typically display a modular organization, with the genes encoding proteins responsible for genome replication, virion morphogenesis, and the structure being compacted into clusters [14]. They replicate via a rolling-circle (RC) mechanism initiated by the phage-encoded replication initiation protein (REP) [13]. In humans, inoviruses are known to contribute to the virulence of pathogenic *Vibrio cholerae* strains [15]. Indeed, the chromosomally integrated CTXϕ prophage encodes a suite of toxins, including the primary cholera toxin (genes *ctxA* and *ctxB*), which causes watery diarrhea. Two additional proteins with enterotoxic activity—the zonula occludens toxin (ZOT) and accessory cholera enterotoxin (ACE)—increase the short-circuit current across rabbit intestinal tissue by altering tight junctions. This allows the passage of macromolecules through mucosal barriers, thereby contributing to *Vibrio cholera* pathogenicity [15]. Whereas CtxAB toxin is not essential for CTXϕ reproduction, ZOT and ACE are indispensable for virion assembly and structure, respectively. In particular, the toxic ZOT peptide is part of the secretion ATPase conserved across all filamentous phages, although the ZOT peptide itself is not [16]. More recently, an additional, unexpected role of filamentous phages in pathogenesis has been demonstrated. Inovirus Pf, secreted by *Pseudomonas aeruginosa*, triggers maladaptive innate viral pattern-recognition responses, which impair bacterial clearance, promoting *P. aeruginosa* skin and lung infections in mice and humans [17,18,19].

In this study, we present the identification and characterization of a new inovirus, named RIP1. The RIP1 genome was detected using high-throughput DNA sequencing from samples collected from blood serum in absence of concomitant bacterial infection or contamination, suggesting possible phage persistence in the human blood.

## 2. Materials and Methods

Human samples

Sera samples (n = 11) were purchased from the “Etablissement Français du Sang” Marseille. Sera were pooled prior to DNA extraction.

Viral isolation and high-throughput sequencing

Each sample was centrifuged at low speed to eliminate proteins and cellular debris. The resulting supernatant was collected and filtered through a 0.45 µm filter pore. Virus-like particles (VLPs) were concentrated by ultracentrifugation at 55,000 g for 60 min. The resulting pellet was resuspended in a phosphate-buffered saline solution (PBS) previously filtered at 0.22 µm. Purified VLPs were treated with DNase and RNase to remove any residual host and bacterial DNA, as previously described [20]. Viral DNA was then extracted using the High Pure Viral Nucleic Acid Kit (Roche Applied Science, Seattle, WA, USA) following the manufacturer’s recommendations. The extracted DNA was amplified using the commercial Illustra^TM^ GenomiPhi V2 DNA Amplification Kit (GE Healthcare Life Sciences, Marlborough, MA, USA) to generate sufficient material for shotgun 454 pyrosequencing library preparation. Amplified DNA was purified using the Agencourt AMPure XP-PCR Purification kit (Beckman Coulter, Brea, CA, USA) to remove the enzyme, dNTPs, and primers, and subsequently sequenced on a 454 Life Sciences Genome Sequencer FLX instrument using titanium chemistry (Roche Applied Science). The complete genome sequence of RIP1 was deposited to GenBank under the accession number KF887906.

Bioinformatics

Obtained sequences from 454 pyrosequencing were screened to remove exact and nearly identical duplicates. Duplicate removal was performed by the CD-HIT-454 program available under the CAMERA 2.0 web portal. The mapping of metagenomic reads and de novo assembly were performed using the CLC Genomics Workbench version 4.9 (Qiagen, Germantown MD, USA). Initial classification of obtained reads using BlastN searches identified sequences homologous to *Ralstonia pickettii* 12D chromosome 1 (Acc. Number # NC_012856.1). The mapping of metagenomics reads was performed on this genome, with a minimal overlap length fraction of 0.5 and a minimal similarity of 0.95 as mapping parameters. De novo contig assemblies were performed with a minimum overlap length fraction of 0.5 and a minimum overlap identity of 0.9. Open Reading Frames prediction was performed using Prodigal software version 2.6.3. Blast analyses were performed using RIP1 ORFs as a query. TBlastN searches were performed of metagenomic sequence read archives (SRA) (n = 35), expressed sequence tags (EST) and transcriptome shotgun assembly (TSA) GenBank databases (accessed in the period between February–March 2024). For SRA obtained from Illumina sequencing, Blast algorithm was adjusted for searches of short sequences.

Polymerase chain reaction and sequencing

Standard PCR amplification was performed using Phusion High-Fidelity DNA Polymerase (Thermo Scientific, Norristown, PA, USA). The absence of kit/reagent contamination was verified with the High Pure Viral Nucleic Acid Kit (Roche Applied Science, Seattle, WA, USA) and Illustra^TM^ GenomiPhi V2 DNA Amplification Kit (GE Healthcare Life Sciences, Marlborough, MA, USA) using 0.02 µm filtered PBS buffer as a sample. PCR amplifications were performed using RP1F/RP1R and RP3F/RP3R primers with the following parameters: an initial denaturation step at 98 °C for 30 s, followed by 35 cycles of 98 °C for 30 s, 60 °C for 30 s, and 72 °C for 45 s. The presence of the *Ralstonia pickettii* 16S rRNA gene was verified using Rp-F1/Rp-R1 forward/reverse primers as previously described [21]. To confirm the presence of sequences found in silico, standard PCRs targeting RIP1 ZOT were performed using ZotIntF1/ZotIntR2 primers with the following parameters: an initial denaturation step at 98 °C for 30 s, followed by 35 cycles of 98 °C for 30 s, 52 °C for 30 s, and 72 °C for 20 s. PCR positive samples were sequenced using the BigDye Terminator v1.1 Cycle Sequencing Kit (Life Technologies, Carlsbad, CA, USA) according to the manufacturer’s instructions. Primer sequences are presented in Supplementary Table S1.

RIP1 CDS cloning

ORF1, ORF3, and ORF14 ORFs were cloned in frame with the Flag-tag at the 5′ region in pCS2+ expression vector using the FlagRepF/FlagRepR, FlagResF/FlagResR, and FlagZotF/FlagZotR primers, respectively. For this purpose, PCR amplicons and pCS2+ vectors were digested using XhoI/BamHI restriction enzymes. Ligation was performed using T4 ligase (Promega, Wisconsin WI, USA) following manufacturer recommendations.

Cell culture, transfection, immunofluorescence, and Western blot

Human embryonic kidney (HEK293T) cells were maintained under standard culture conditions (37 °C and 5% CO_2_) and cultured in Minimum Essential Media (MEM) supplemented with 10% Fetal Bovine Serum (FBS), 1% Glutamine, and 1% Penicillin-Streptomycin (PS). Cells were transfected using Lipofectamine 2000 (Life Technologies) following manufacturer specifications. For cell immunofluorescence staining, transfected cells were fixed for 20 min in PFA 4% at 37 °C then washed three times in PBS and incubated for 20 min in blocking buffer (PBS + 0.1% Triton X-100, BSA 3%). Mouse FlagM2 antibodies (Sigma-Aldrich, Louis, MO, USA) diluted at 1/200 in blocking buffer were incubated for 1 h at room temperature followed by three washes in PBS + 0.1% Triton X-100. Secondary anti-mouse Alexa 488 antibody (1/1000) was incubated for 1 h. Cell nuclei were stained with ProLong Gold Antifade reagent (Molecular Probes, Eugene, OR, USA) containing DAPI. For Western blot, cell proteins were extracted in radioimmunoprecipitation assay (RIPA) buffer (1% NP-40, 0.5% deoxycholic acid, 0.1% SDS in PBS, containing protease inhibitors). Proteins were resolved in a 12% SDS-PAGE gel and transferred onto a 0.2 µm nitrocellulose membrane and then immunoblotted using mouse FlagM2 antibody (Sigma-Aldrich, Louis, MO, USA) diluted at 1/500.

## 3. Results

### 3.1. The Identification of a New Inovirus in the Human Blood

**Figure 1:**
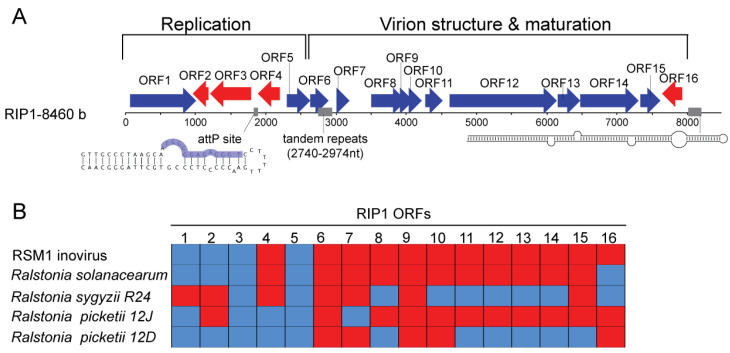
Identification of a new inovirus RIP1. (**A**) Linear representation of the genomic organization of the RIP1 element. The length in bases (b) is given on the left. Forward/reverse ORFs are represented with blue/red arrows, respectively. The presence of *cis* elements is outlined with grey boxes. The secondary structure of the *attP* site and the long hairpin, corresponding to the predicted genome packaging signal, are represented below the linearized sequence. The core sequence of the *attP* site is outlined in blue. (**B**) The presence/absence of homologous genes in *Ralstonia picketii* 12D, *Ralstonia sygyzii* R24, *Ralstonia picketii* 12J, *Ralstonia solanacearum* CRM15, and RSM1 phage genomes is reported using blue/red boxes, respectively.

In the course of the study of virome composition in the human blood using a viral metagenomics approach [22], we detected the presence of metagenomic reads homologous to the ssDNA *Ralstonia solanacearum* phages RSM1 and RSM3 (later referred to as RSM 1/3), which are members of the genus Habenivirus in the Inoviridae family. The de novo genomic assembly of the blood metagenome revealed that these reads organized a single contig of 8.5 kb in length with 16 predicted open reading frames (ORF) (Figure 1A). We identified several cis-regulatory elements typical of inoviruses present in the RIP1 genome. Among these, we detected a conserved core sequence (between 1846 and 1858) of the attachment site attP required for the integration of the phage genome into the host chromosome with the aid of phage-encoded serine superfamily integrase, previously functionally characterized in RSM 1/3 phages [23] (Figure 1A). At the 3′ end of the contig, we identified a long hairpin (Figure 1A), which potentially corresponds to the phage packaging signal typically found in filamentous phages [13,16]. Notably, we also detected a tandem repeat sequence containing only one internal mismatch at position 2740–2974 with an unknown function.

The majority of RIP1 ORFs coded for hypothetical proteins and had their homologous counterparts in the RSM 1/3 viral genomes, as well as in the inovirus-like prophage genomes integrated in the bacterial chromosomes of the Ralstonia group (Figure 1B and Figure S1). Thus, we named this metagenomic contig RIP1 (Ralstonia Inoviridae Phage 1). BlastP analyses and a thorough comparison of RIP1 ORF products with those encoded by other filamentous phages allowed for the identification of all structural proteins typical of inoviruses among the gene products of RIP1 (Table 1). Indeed, ORF11 encodes for the pVIII-like (here and elsewhere the protein nomenclature of M13-like inoviruses is used) viral major capsid protein, which organizes into a helical array covering the phage DNA and forming the virion. RIP1 ORF11 protein contains the characteristic N-terminal signal sequence, followed by the amphipathic, hydrophobic, and basic domains (Table 1; Figure S2A) essential for positioning of the protein in the membrane prior to virion assembly, the tight packing of the capsid proteins in the virion tube following the assembly, and the interaction with the phage DNA within mature virions [11]. RIP1 ORF9 and ORF10 were, respectively, identified as the homologs of pVII and pIX minor capsid proteins of M13-like inoviruses. The two proteins are located at the tip of the filamentous phage particle, which is the first to emerge during virion assembly; both are small, roughly 3–3.5 kDa, membrane proteins and possess one transmembrane domain each (Table 1; Figure S2B,C). ORF12 and ORF13 are homologous to the pIII-like and pVI-like (Figure S2D) inoviral proteins, which mediate virion assembly termination, release, and infection. The pIII homologue in RIP1 was identified after several PSI-BLAST iterations using the ORF12 sequence as a query. ORF5 corresponds to pV, an OB-fold domain protein which binds to the single-stranded form of the genome, thereby controlling the switch from the double-stranded replicative intermediate to the synthesis of the (+) stand, which is subsequently packed into the progeny virions. RIP1 ORF14 corresponds to pI, a homolog of the ZOT toxin encoded by the CTXϕ phage. However, the C-terminal region of the CTXϕ ZOT protein responsible for its activity as a toxin (288–293 aa) was lacking in the RIP1 ORF14, suggesting that ORF14 exclusively functions in virion assembly, without eliciting toxicity.

### 3.2. RIP1 ORF1 and ORF3 Display Nuclear Subcellular Localization

**Figure 2:**
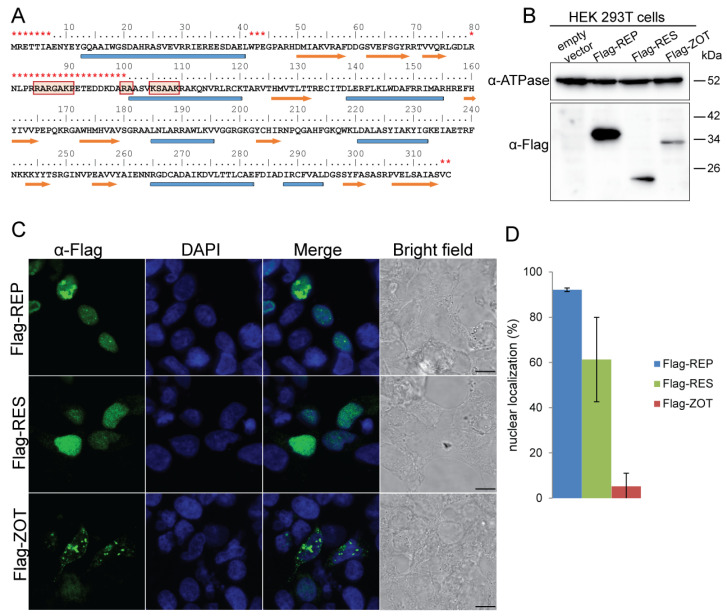
RIP1 REP and RES proteins have nuclear localization in human cells. (**A**) Primary and secondary structure of the REP protein. The predicted nuclear localization signal is highlighted with red boxes. Alpha helices are represented with blue cylinders, beta sheets are represented with orange arrows, and unstructured regions with orange asterisks. (**B**) Western blot using anti-Flag antibody showing the expression of Flag-tagged REP, RES, and ZOT proteins. Anti-ATPase antibody was used for calibration purposes. (**C**) Confocal images showing the subcellular localization of Flag-tagged REP, RES, and ZOT proteins. Nuclei were stained using DAPI. Scale bars; 2 µm. (**D**) Histogram presenting the percentage of cells with an anti-Flag fluorescence signal co-localization with DAPI nuclear dye (mean ± s.d.; three independent experiments).

Next, we focused on the RIP1 ORF1 and ORF3. ORF1 encodes for the REP homolog required for phage DNA replication via a rolling-circle (RC) mechanism. All three signature motifs found in the HUH superfamily RC-REP proteins of bacterial, archaeal, and eukaryotic ssDNA viruses [24,25] were conserved in the product of ORF1 (Figure S3). ORF3 encodes the resolvase-like serine recombinase (RES) required for phage integration into the bacterial genome (Figure S4). Notably, the integration/excision activity of the close homologue of RIP1 ORF3 from the RSM1 phage (80% identity over 100% of alignment) has been demonstrated experimentally [26]. Unexpectedly, using the NLStradamus web-based interface [27], we detected a eukaryotic nuclear localization signal (NLS) in the REP protein (Figure 2A). To confirm this observation, we expressed the REP protein in human embryonic kidney (HEK) cells. Immunofluorescence experiments indeed detected the REP protein in the nucleus (Figure 2B,C). Furthermore, the RES protein also showed similar subcellular localization, although NLS was not detected in its primary structure (Figure 2C). Notably, nuclear localization was not systematic for all RIP1 proteins. Indeed, the expression of the pI/ZOT protein (ORF14), required for phage assembly in HEK cells, resulted in cytoplasmic localization, suggesting that the nuclear targeting is restricted to the phage DNA replication/recombination machinery.

### 3.3. RIP1 Prevalence in the Human Blood Is Not Concomitant with a Bacterial Infection

It has been previously reported that genomes of ssDNA viruses (although not filamentous phages) are occasionally detected in human samples, but instead of being associated with infection, they were subsequently traced to contaminations of the DNA extraction spin columns [28,29]. The formal possibility that the presence of the RIP1 genome in human samples was due to contamination was excluded by the systematic testing of all kits and reagents used in this study for DNA isolation and amplification by polymerase chain reaction (PCR). This analysis showed that none of the reagents or kits contained RIP1 DNA. Furthermore, the presence of RIP1 in the viral metagenomes was verified by performing PCR using ORF14-specific primers on viral DNA extracted from sera samples. Notably, metagenomics analysis, as well as pan-genomic 16S rRNA and *Ralstonia pickettii*-specific 16S rRNA PCRs, did not reveal a bacterial presence in these samples.

We further performed tBlastN searches against metagenomic sequence read archive (SRA), expressed sequence tags (EST) and transcriptome shotgun assembly (TSA) GenBank databases using RIP1 ORFs as query. We were able to identify sequences homologous to RIP1 ORFs in human metagenomes (n = 35) generated from diverse anatomical sites, including blood, amniotic fluids, feces and nasal swabs (Table S1). Interestingly, the majority of sequences mapped to REP and RES ORFs. Importantly, additional tBlastN searches against expressed sequence tags (EST) and transcriptome shotgun assembly (TSA) databases, using RIP1 ORFs as queries, identified transcripts corresponding to the ORF1, ORF2, and ORF3 in other animals, including *Sus scofa*, *Schistosoma mansoni*, and *Tupaia chinensis*, suggesting that the presence of RIP1-related viruses is not restricted to humans (Table S1).

## 4. Discussion

Here, we present the identification of a new bacteriophage named RIP1 in the human blood. Genome organization, as well as genetic composition, strongly suggest that RIP1 is a member of the *Inoviridae* family. All the components required for the assembly and structure of M13-like inoviruses are conserved in the RIP1 genome (Figure S2). Based on a comparison of the RIP1-related sequences, it appears that it shares its most recent common ancestor with proviruses integrated in the genomes of *Ralstonia* species (Figure 1B). Notably, in all three *Ralstonia* elements, the ZOT-like proteins lack the toxigenic peptide found in the C-terminal region of the ZOT protein of the *Vibrio cholera* phage CTXϕ [30], suggesting that the proteins of *Ralstonia* phages are unlikely to elicit adverse effects on the tight junctions.

A comparative genomic analysis of the RIP1 genome revealed that it is a mosaic of genes derived from various inoviruses (Figure S1). Indeed, genomes of the *Inoviridae* members are known to be shaped by frequent recombination events, which often leads to non-orthologous gene replacements and the acquisition of new genes [14]. Thus, the RIP1 ancestor has apparently emerged as a result of recombination between an RSM1-like phage, which has donated the genome replication/recombination modules, and a phage that contributed to the virion structure and assembly module.

RIP1 genome was isolated from a limited number of sera samples obtained from blood pockets, thus the estimation of RIP1 prevalence in the general population remains unknown and should be further addressed. The occurrence of inoviral-related sequences in multiple human samples points towards temporal phage persistence after asymptomatic infection. Strikingly, we found that the abundance RIP1 sequences, represented up to 10% of all metagenomics reads. However, viral metagenome from blood was amplified using phi29 DNA polymerase, which displays a preference towards circular ssDNA matrices [31], thus rendering RIP1 abundance estimation difficult. The most likely route for RIP1 emergence in the human virome is via its *Ralstonia* host. Human-associated *Ralstonia pickettii* were found to be part of the commensal flora of the oral cavity and the upper respiratory tract of healthy individuals [21,32]. Furthermore, *Ralstonia pickettii* can be isolated from a variety of clinical specimens, including sputum, blood, wound infections, urine, ear and nose swabs, and cerebrospinal fluids [33]. In this study, 16S rRNA gene PCRs were unable to identify the presence of bacterial infection in our samples, thus strongly suggesting that RIP1 detection is not due to bacterial contamination. Alternatively, the phage could have evolved to infect a different bacterium from the human microbiome. Another possibility, even though highly speculative, is that the REP- and/or RES-mediated introduction of the RIP1 genome into the nucleus could lead to the occasional proliferation of RIP1 in the human genome in the form of a mobile genetic element.

Bacteriophages are generally not considered to have any direct effect on eukaryotes. However, it has been shown that bacteriophages are capable of rapid and directional transcytosis across confluent cell layers originating from the gut, lung, liver, kidney, and brain, accessing both the vesicular and cytosolic compartments of the eukaryotic cells [34]. It has been concluded that the transcytosis of bacteriophages is a natural and ubiquitous process that provides a mechanistic explanation for the occurrence of phages within the body. Furthermore, it has been demonstrated that terminal proteins, which are covalently attached to the termini of the genomes of certain dsDNA bacteriophages, contain functional NLS [35,36]. This finding has suggested that the genomes of these bacteriophages have access to the eukaryotic nucleus and might facilitate inter-domain horizontal gene transfer. In the present study, we have shown that proteins of an ssDNA inovirus that are involved in DNA metabolism, including replication initiation (RC-REP) and recombination (RES), can also enter the eukaryotic nucleus. Importantly, upon replication initiation, RC-REPs form a covalent intermediate with the viral DNA, suggesting that, like in the case of terminal protein-encoding bacteriophages [35], RIP1 REP might shuttle the inoviral DNA into the nucleus. This opens an intriguing possibility that RIP1 proteins might elicit their corresponding enzymatic activities in proximity of eukaryotic genomes. For example, RES could mediate recombination between the phage and cellular chromosomes. Notably, serine recombinases are widely used in genetic engineering for the stable integration of transgenes into eukaryotic cells [37]. Although inovirus-like sequences have not been reported in eukaryotic genomes, many eukaryotes contain endogenized fragments of eukaryotic ssDNA viruses [38,39].

Interestingly, another RIP1 protein with functional NLS is homologous to the RC-REPs widespread in prokaryotic and eukaryotic ssDNA viruses and transposons (Figure S3). In this context, it is worth mentioning that eukaryotic rolling-circle transposons of the Helitron family have been reported to show closer a similarity to REPs encoded by inoviruses and bacterial plasmids than to bacterial transposases or eukaryotic ssDNA viruses [40]. It is thus tempting to speculate that prokaryotic ssDNA viruses, such as RIP1, might give (or even have given) rise to new classes of eukaryotic transposable elements. Additionally, the recombination between different groups of ssDNA viruses has played a key role in the evolution of this virus realm [25]. The co-localization of prokaryotic and eukaryotic ssDNA viruses thus provides additional opportunities for exploring the genetic landscape for generating novel recombinant virus types. Obviously, further studies will be required to clarify the origin, function, and impact of RIP1 in the human virome.

## Figures and Tables

**Table 1:** Summary information on the predicted ORFs of the RIP1 phage. From the left to the right, ORF number, location, orientation, corresponding protein length, conserved domains, and predicted functions are reported. The research of conserved domains and signatures was performed using CDD, PFAM, and Prosite databases.

ORF#	Position (Bases)	Strand	Protein Length	Conserved Domains	Predicted Functions
1	62–1012	+	316	NOT FOUND	rolling circle DNA replication initiation (pII-like)
2	963–1187	−	74	NOT FOUND	Unknown (Hypothetical protein)
3	1203–1793	−	196	Resolvase, N terminal domain (2–140 aa); Helix-turn-helix domain (148–184 aa)	DNA recombination (Resolvase-like)
4	1895–2197	−	100	Helix-turn-helix domain (16–61 aa); DUF4072 domain (18–47 aa)	DNA binding
5	2302–2631	+	109	Helix-destabilising domain (10–35 aa); Phage DNA bind	ssDNA binding (pV-like)
6	2628–2894	+	88	NOT FOUND	Protein folding, Chaperone (Highly divergent DNA J-like protein)
7	3002–3190	+	62	AA transfer class 2 attachment site (20–29 aa)	Unknown (Hypothetical protein)
8	3495–4001	+	168	GrpE family [PF01025] (21–104 aa)	Protein folding, Chaperone (GrpE-like)
9	4012–4098	+	28	Hydrophobic domain (5–27)	Phage assembly, minor capsid protein (pVII-like)
10	4095–4214	+	39	Transmembrane domain (11–32 aa)	Phage assembly, minor capsid protein (pIX-like)
11	4272–4523	+	83	N-terminal signal sequence (30), amphiphatic (40–54), hydrophobic (55–71) and basic domains (72–83)	Phage assembly (pVIII-like)
12	4617–6137	+	506	Threonine-rich region (303–364 aa)	Termination of virion assembly (pIII-like)
13	6150–6479	+	109	Hydrophobic domains (4–20; 33–55; 75–97)	Termination of virion assembly (pVI-like)
14	6481–7311	+	276	Zonular occludens toxin (ZOT) domain	Phage assembly (pI-like)
15	7334–7618	+	94	NOT FOUND	Unknown (Hypothetical protein)
16	7640–7927	−	95	NOT FOUND	Unknown (Hypothetical protein)

## Data Availability

Data are contained within the article and Supplementary Materials.

## Supplementary Materials

The following supporting information can be downloaded at: https://www.mdpi.com/article/10.3390/v16030475/s1, Table S1: tBlastN searches against SRA, EST and TSA databases using RIP1 ORFs; Table S2: Summary table of primers used in this study; Figure S1: RIP1 has a chimeric genomic organization; Figure S2: Multiple alignments between RIP1 ORFs and homologous inovirus proteins; Figure S3: RIP1 ORF1 encodes a rolling-circle replication initiation protein; Figure S4: RIP1 ORF3 encodes a serine recombinase.
