# Supplementary material for: A New Inovirus from the Human Blood Encodes Proteins with Nuclear Subcellular Localization
Source: Viruses. 2024 Mar 20;16(3):475. doi: 10.3390/v16030475 (PMC10975378; doi:10.3390/v16030475)
Supplement: Supplementary file 1 [file viruses-16-00475-s001.zip › viruses-2825377-supplementary.pdf]

Supplementary Information

A new inovirus from the human blood encodes proteins with nuclear subcellular localization

Nikolay Popgeorgiev[1, a*], Mart Krupovic[2], Julien Hiblot[3], Laura Fancello[4], Sonia Monteil-Bouchard[5], and Christelle Desnues[5,†]

TableS1: tBlastN searches against SRA, EST and TSA databases using RIP1 ORFs.

**Organism name/ Biosample**	**Origin**	**Accession #**	**Sequencing technique**	**SRA, EST, TSA Accession #**	**Sequences related to RIP1 ORFs:**	**Number of sequences detected/ E-value**
**NCBI SRA metagenomic database**						
Homo sapiens/ Serum	Japan	SAMD00008984	Illumina	DRX000952	1,3,7,15	7
Homo sapiens /Nasopharyngeal swabs	Kazakhstan	SAMN36835585	Ion Torrent	SRX21246575, SRX21246574, SRX21246571, SRX21246569, SRX21246568, SRX21246565, SRX21246535, SRX21246557	1,3,5,9, 10,12,14	152
Homo sapiens /Stool	China	SAMN28109099	Illumina	SRX15540575, SRX15540587	3	>200
Homo sapiens /Amniotic fluids	China	SAMN34062693 SAMN34062696	Illumina	SRX19859627, SRX19859630	1,3	38
**NCBI EST and TSA databases**						
Sus scrofa / 4-Cell-Embryo	USA	SAMN00174820	cDNA	CN025072.1	1	$8e^{-19}$
Schistosoma mansoni/ mixed pool	Brasil	SAMN00172663	cDNA	CF497648.1	1,2,3	$5e^{-48}$
Tupaia chinensis/ liver	China	SAMN00794552	TSA/Illumina	JU128735.1	3	$2e^{-43}$

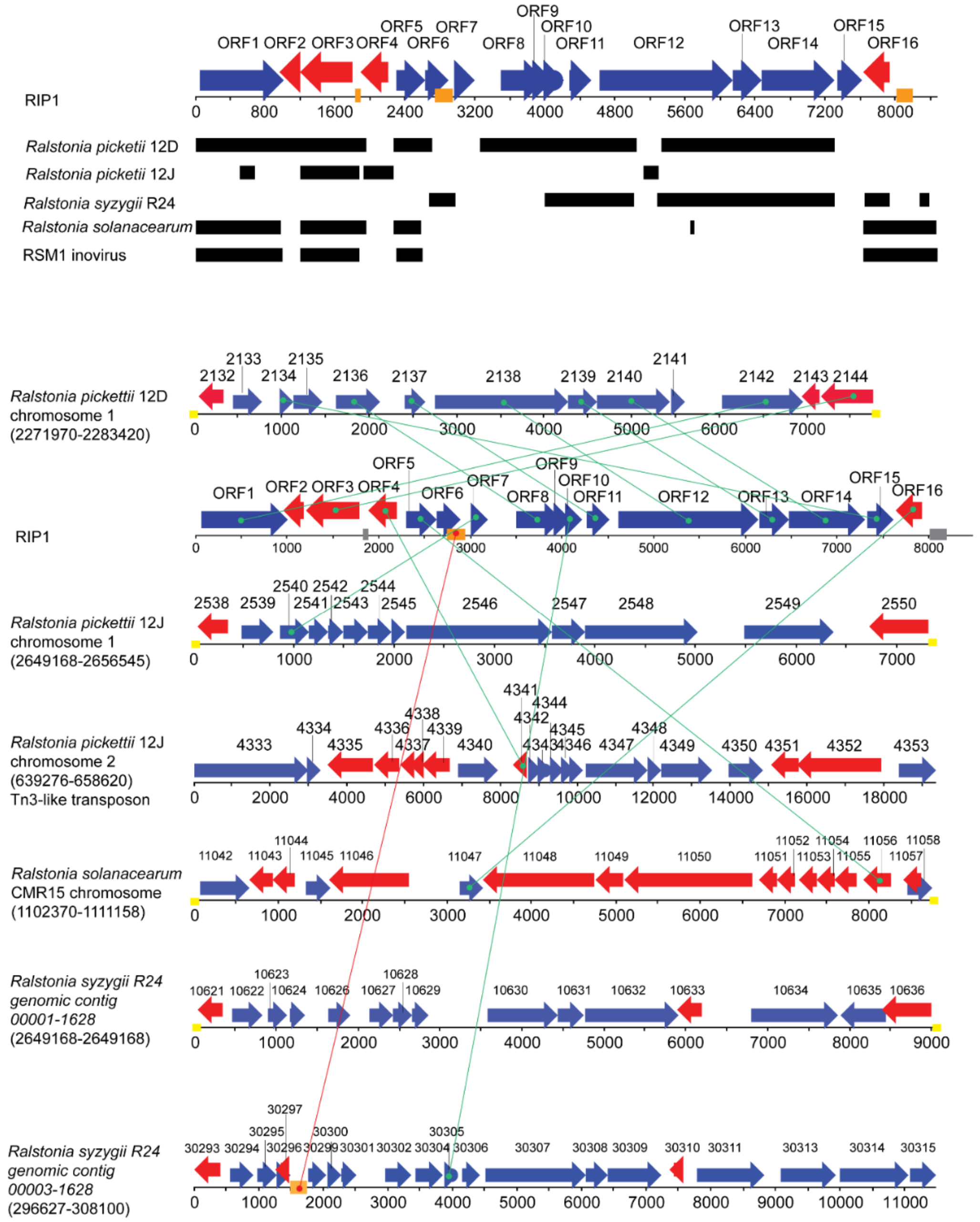

Figure S1: RIP1 has a chimeric genomic organization.

(A) Schematic representation of local alignment results between RIP1 genome and genomes of *Ralstonia picketii 12D*, *Ralstonia sygyzii R24*, *Ralstonia picketii 12J*, *Ralstonia solanacearum CRM15* and RSM1 phage. The matching regions as well as their locations on the RIP1 genome are reported using black dashes. (B) Genomic organization comparison between RIP1 genome and homologous bacterial regions. Forward/reverse ORFs are represented with blue/red arrows respectively. The presence of *attP* sites are represented with yellow boxes. Best Blast hits correspondence between RIP1 ORFs and bacterial ORFs are represented with colored lines.

The presence of *cis* elements are outlined with beige boxes. DNA sequence homologous to *Ralstonia sygyzii R24* non coding region located inside ORF6 is reported with orange boxes.

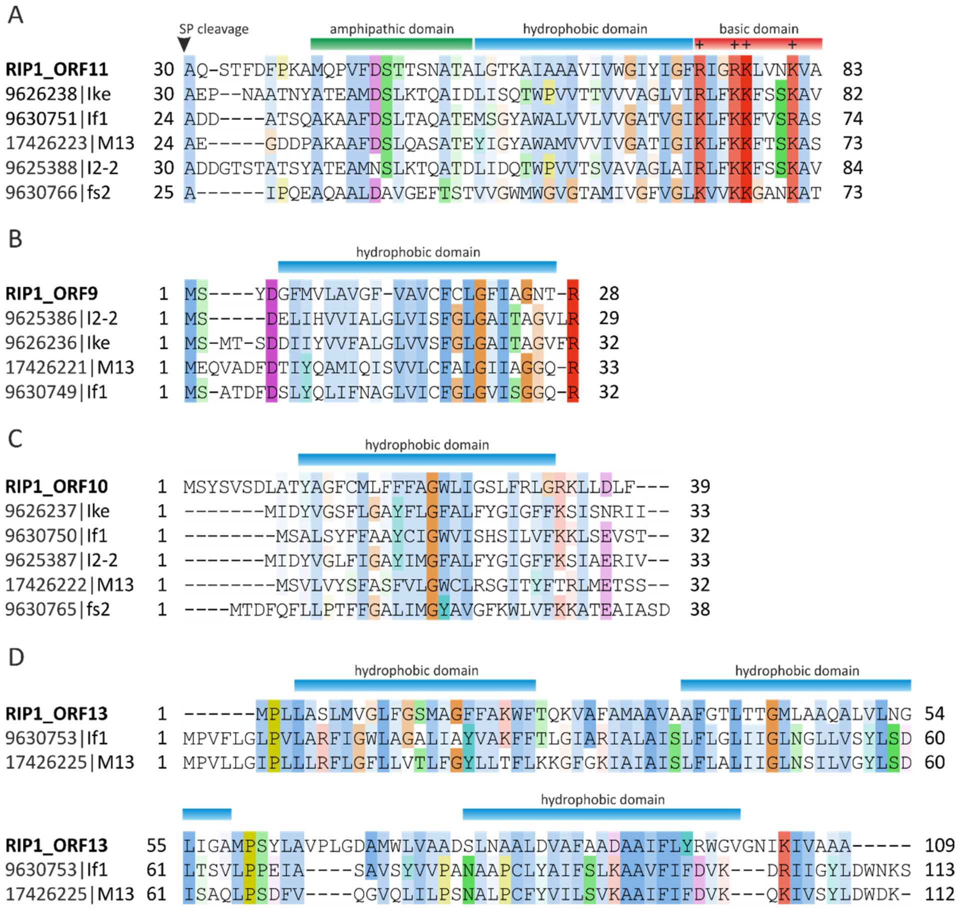

Figure S2: Multiple alignments between RIP1 ORFs and homologous inovirus proteins. (A) Multiple sequence alignment between ORF9 and pVII homologs of inoviruses. The location of a single hydrophobic domain is represented with a blue bar. **(B)** Multiple sequence alignment between ORF10 and pIX-like proteins of inoviruses. **(B)** Multiple sequence alignment between ORF11 and pVIII-like inoviral homologs. The amphiphatic, hydrophobic and basic domains are represented with green, blue and red bars, respectively. Positively charged residues are indicated with (+). (**D**) Multiple sequence alignment between ORF13 and pVI-like inoviral homologs. Abbreviations: SP, signal peptidase; Ike- Enterobacteria phage Ike; If1- Enterobacteria phage If1; I2-2- Enterobacteria phage I2-2; M13- Enterobacteria phage M13; fs2- Vibrio phage fs2. GenBank identifiers for the depicted proteins are provided in the figure.

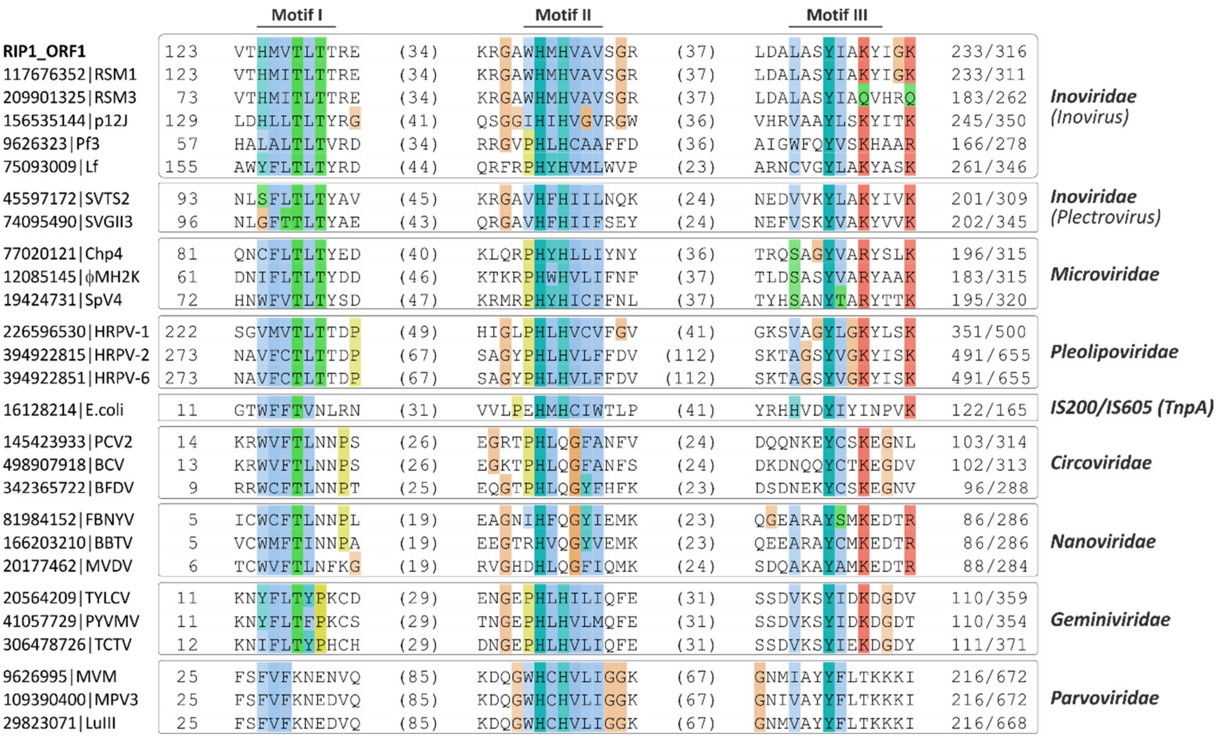

Figure S3: RIP1 ORF1 encodes a rolling-circle replication initiation protein.

Alignment of the RC-REP protein encoded by RIP1 with the corresponding proteins encoded by ssDNA viruses infecting bacteria (*Inoviridae* and *Microviridae*), archaea (*Pleolipoviridae*), and eukaryotes (*Circoviridae, Nanoviridae, Geminiviridae and Parvoviridae*) as well as TnpA transposase of the IS200/IS605 family from *E. coli*. The protein sequences are denoted by their GenBank identiers, followed by the corresponding virus names. Only the three conserved motifs (I–III) typical of the RC-REPs are shown. The limits of the depicted motifs are indicated by the residue positions on each side of the alignment, while the number following the slash sign corresponds to the total length of the protein. The numbers in the parenthesis within the alignment indicate the distance between the motifs.

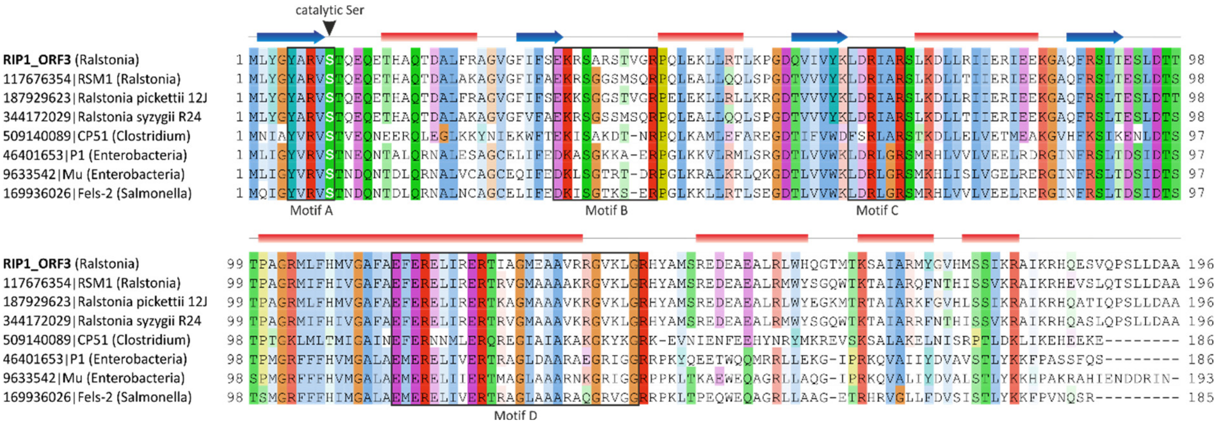

Figure S4: RIP1 ORF3 encodes a serine recombinase.

Alignment of the RIP1 ORF3 product sequence with the corresponding sequences of serine recombinases encoded by various prophages and bacteriophages. The four conserved motifs (A–D) typical of serine recombinases are boxed, while the position of the catalytic serine residue is indicated with an arrowhead. The predicted secondary structure elements (α-helixes, red rectangles; β-strands, blue arrows) are shown above the alignment. Proviruses are indicated by the names of the organisms in which they reside, while the host names of the bacteriophages are provided in parenthesis. GenBank identifiers for all depicted proteins are provided in the figure.

TableS2: Summary table of primers used in this study.

Primer	Sequence (5'-3')	Used in
**FlagRepF**	ATATGGATCCATGGACTACAAGGACGACGATGACAAG ATGCGTGAAACTACGATAGCAG	This study
**FlagRepR**	ATATCTCGAGTCAACAAACACTGGCTATGGC	This study
**FlagResF**	ATATGGATCCATGGACTACAAGGACGACGATGACAAG ATGTTGTACGGTTACGCCCG	This study
**FlagResR**	ATATCTCGAGTTACGCGGCGTCTAGCAAGC	This study
**FlagZotF**	ATATGGATCCATGGACTACAAGGACGACGATGACAAG ATGCCGATCTACATCATCACGG	This study
**FlagZotR**	ATATCTCGAGTCACCATGAGGGGCATCGAG	This study
**Rp-F1**	ATGATCTAGCTTGCTAGATTGAT	[21]
**Rp-R1**	ACTGATCGTCGCCTTGGTG	[21]
**ZotIntF1**	TGTGGATACGTTCACGCTCG	This study
**ZotIntR2**	CGTTCGTCCAGTTTCGCTTC	This study
**RP1F**	CCCGCAAGCGCGCCAACGCT	This study
**RP1R**	ACGATCGGGATTGGTATCGC	This study
**RP3F**	AAACAGTGCGTCGGTTTGTG	This study
**RP3R**	ATCACGCGAGACGAACAACT	This study
